# Deinbollia mosaic virus: a novel begomovirus infecting the sapindaceous weed *Deinbollia borbonica* in Kenya and Tanzania

**DOI:** 10.1007/s00705-016-3217-9

**Published:** 2017-01-09

**Authors:** Martina Kyallo, Peter Sseruwagi, Robert A. Skilton, Mildred Ochwo-Ssemakula, Peter Wasswa, Joseph Ndunguru

**Affiliations:** 1 Biosciences Eastern and Central Africa-International Livestock Research Institute (BecA-ILRI) Hub, P.O. Box 30709-00100, Nairobi, Kenya; 2 Mikocheni Agricultural Research Institute, P.O. Box 6226, Dar es Salaam, Tanzania; 3 School of Agricultural Sciences, Makerere University, P.O. Box 7062, Kampala, Uganda; 4 International Centre of Insect Physiology and Ecology (icipe), P.O. Box 30772-00100, Nairobi, Kenya

## Abstract

Four isolates of a bipartite begomovirus from naturally infected *Deinbollia borbonica* plants exhibiting yellow mosaic symptoms in Kenya and Tanzania were molecularly characterised. The DNA-A was most closely related to that of tomato leaf curl Mayotte virus (AM701764; 82%), while the DNA-B shared the highest nucleotide sequence identity with that of East African cassava mosaic virus (AJ704953) at 65%. Based on the current ICTV species demarcation criterion for the genus *Begomovirus* (≥91% sequence identity for the complete DNA-A), we report the full-length genome sequence of this novel bipartite begomovirus. The results reveal additional diversity and reservoir hosts of begomoviruses in East Africa.

**Electronic supplementary material:**

The online version of this article (doi:10.1007/s00705-016-3217-9) contains supplementary material, which is available to authorized users.

Begomoviruses (family *Geminiviridae*) are an extremely successful group of emerging viruses infecting cultivated (crop), and non-cultivated (weed) plants from different botanical families [1]. The high degree of genetic variability and species diversity exhibited by begomoviruses in Africa threatens the cultivation of economically important crops such as cassava, tomato and beans [2]. Weed-infecting begomoviruses are transmitted by the polyphagous *Bemisia tabaci* (Gennadius) (Aleyrodidae) [3] and may act as progenitors of crop-infecting begomoviruses [4] or increase the genetic diversity of the latter by recombination [5]. Despite the critical role that weed-infecting begomoviruses play in the epidemiology of crop diseases, they remain under-studied in Africa.

*Deinbollia borbonica* Scheff. (Sapindaceae) is a common perennial tropical shrub whose geographical distribution spreads from the coastal belt of Somalia to northern Mozambique. In East Africa, it grows as a weed within mixed cropping farming systems where crops such as cassava, tomato, and beans are grown. Despite *D. borbonica* existing in Africa for over a century (https://plants.jstor.org/stable/10.5555/al.ap.specimen.m0108980), it was not until in 2012 that a begomovirus naturally infecting *D. borbonica* was identified in northeastern Tanzania by Ndunguru et al. (unpublished; GenBank accession no. KT799138). Here, we report the full-length genome sequence of this novel bipartite begomovirus infecting *D. borbonica* in Kenya and Tanzania.

Infected leaf samples showing yellow mosaic symptoms (Supplementary Fig. S1) were collected from *D. borbonica* in Kenya (sample K1; GPS coordinates 03.31669S, 39.96528E; altitude 12 masl; May 2014) and Tanzania (samples T1, T2, and T3; GPS coordinates 05.10219S, 38.47172E; altitude 202 masl; March 2015). Total DNA was extracted using a ZR Plant/Seed DNA MiniPrep Kit (Zymo Research Corp.). The identity of the plant species was confirmed by PCR amplification and Sanger sequencing of the chloroplast *rbcL* gene [6, 7]. The virus genome was enriched by rolling-circle amplification (RCA) using a TempliPhi 100 RCA Kit (GE Healthcare), and sequenced using an Illumina MiSeq system at BecA-ILRI Hub (Nairobi, Kenya). *De novo* assembly using CLC Genomics Workbench version 7.0.4 (CLC Bio; QIAGEN) generated two consensus contigs identified as DNA-A and DNA-B by BLASTn analysis (http://www.ncbi.nlm.nih.gov/BLAST). The genome components were amplified using abutting primers (DNA-Af, 5′-TTGGGCTCCAAGTTTTGACG-3′; DNA-Ar, 5′-TACGCGTCAAAACTTGGAGC-3′) and DNA-Bf, 5′-TGTCGTATGCGTGCTTTTGG-3′; DNA-Br, 5′-AATAGCCTCCAAAAGCACGC-3′) using Phusion High-Fidelity DNA Polymerase (Thermo Fisher Scientific). The blunt-ended PCR products were cloned into PUC18 and sequenced by the Sanger method using overlapping PCR primers (Supplementary Table S1).

The genome organization of the virus was identical to that of Old World bipartite begomoviruses (Supplementary Table S2). The DNA-A and DNA-B contained a 158-nucleotide (nt)-long common region (CR) with >97 % nucleotide sequence identity. Additionally, a unique putative iteron sequence (GAGGGCA), appearing twice as perfect repeats and once as an inverted repeat sequence, was identified, indicating that the cloned DNA-A and DNA-B from each plant sample constituted a cognate pair. The CR also possessed the nonanucleotide sequence (5′-TAATATTAC-3′) typical of begomoviruses [8]. Recombination analysis using RDP4 revealed no evidence of significant recombination events.

Pairwise nucleotide sequence identities calculated using Sequence Demarcation Tool (SDT v1.2) [9] revealed that the isolates were >98% identical to each other and <91% identical to currently reported begomoviruses (Supplementary Fig. S2). According to the recently updated ICTV begomovirus species demarcation criteria [10], the isolates qualify to belong to a novel begomovirus species, tentatively named “*Deinbollia mosaic virus*” (based on the host plant and disease symptom). Phylogenetic reconstruction based on the full-length nucleotide sequences of the DNA-A (Fig. 1A) and DNA-B (Fig. 1B) using GTR + G + I as the best-fit model of evolution in MEGA 6 software [11], clustered DMV isolates together with African begomoviruses. The DNA-A sequence was most similar (82%) to that of tomato leaf curl Mayotte virus (ToLCYTV; AM701764), while the DNA-B sequence shared 65% nucleotide sequence identity with that of East African cassava mosaic virus (EACMV: AJ704953). The results reveal additional diversity and reservoir hosts of begomoviruses in East Africa.

**Fig. 1:**
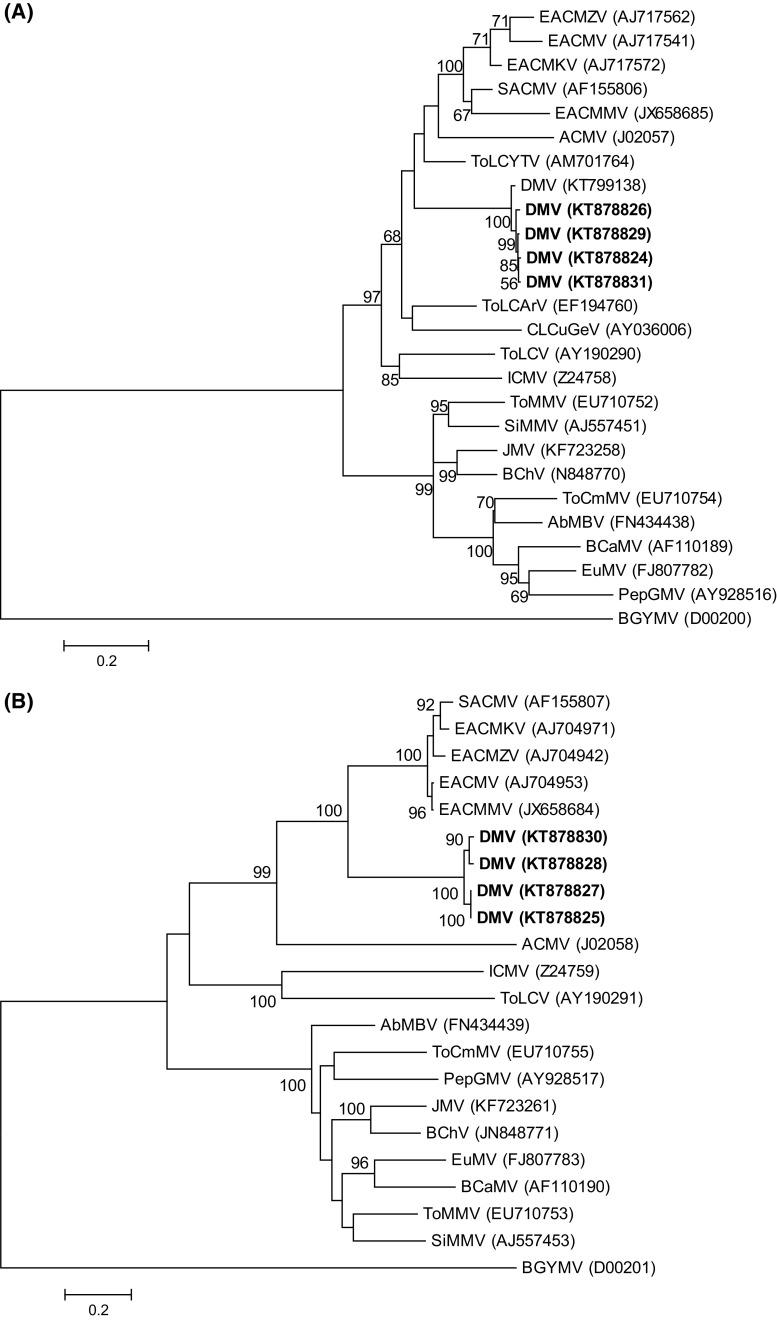
Maximum-likelihood (ML) trees inferred from the alignment of full-length DNA-A (**A**) and DNA-B (**B**) nucleotide sequences of DMV (in bold) and selected begomoviruses. Bootstrap values above 50% (1,000 replications) are indicated above the branches. Refer to Supplementary Table S3 for begomovirus names and acronyms

**GenBank accession numbers**

KT878823, KT878824, KT878825, KT878826, KT878827, KT878828, KT878829, KT878830, KT878831.

## Electronic supplementary material

Below is the link to the electronic supplementary material.

**Supplementary Fig. S1** Healthy (**A**) and naturally infected (**B**) *Deinbollia borbonica* plants. The infected plant shows yellow mosaic symptoms and distortion of leaves (PDF 184 kb)

**Supplementary Fig. S2** Nucleotide sequence identity plot of the full-length DNA-A of DMV and reference begomoviruses calculated using Sequence Demarcation Tool (SDT) v. 1.2 (PDF 43 kb)

**Supplementary Table** **S1** (PDF 145 kb)

**Supplementary Table** **S2** (PDF 53 kb)

**Supplementary Table** **S3** (XLSX 13 kb)
