# Supplementary figures and images for: Deinbollia mosaic virus: a novel begomovirus infecting the sapindaceous weed Deinbollia borbonica in Kenya and Tanzania
Source: Arch Virol. 2017 Jan 9;162(5):1393–6. doi: 10.1007/s00705-016-3217-9 (PMC5387033; doi:10.1007/s00705-016-3217-9)

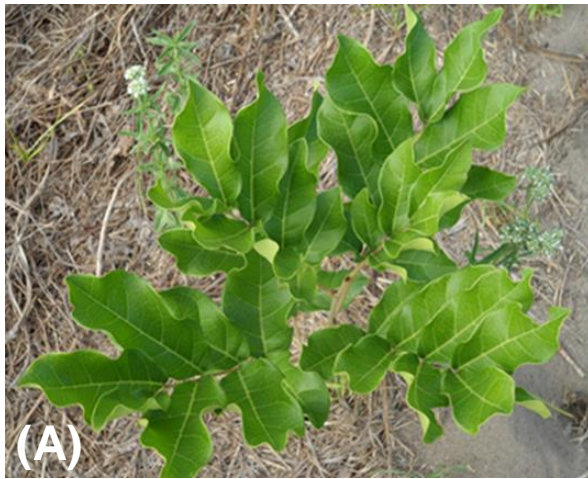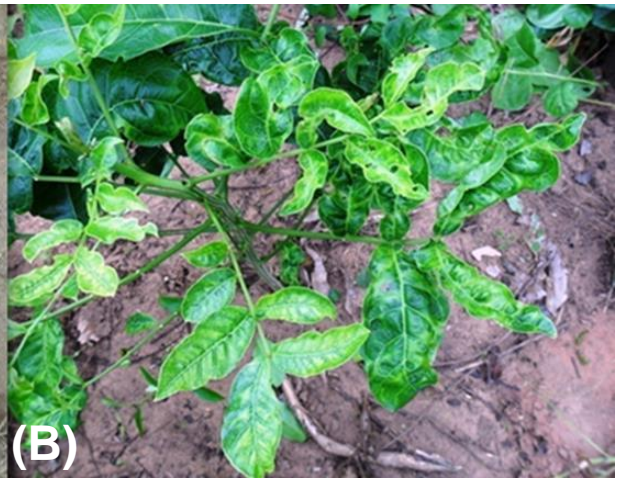

Supplement: Supplementary file 1 — Supplementary Fig. S1 Healthy (A) and naturally infected (B) Deinbollia borbonica plants. The infected plant shows yellow mosaic symptoms and distortion of leaves (PDF 184 kb) [file 705_2016_3217_MOESM1_ESM.pdf]

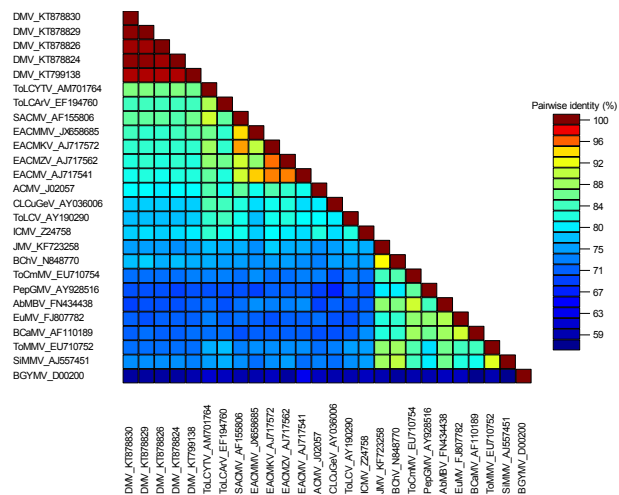

Supplement: Supplementary file 2 — Supplementary Fig. S2 Nucleotide sequence identity plot of the full-length DNA-A of DMV and reference begomoviruses calculated using Sequence Demarcation Tool (SDT) v. 1.2 (PDF 43 kb) [file 705_2016_3217_MOESM2_ESM.pdf]
