# Supplementary material for: Deinbollia mosaic virus: a novel begomovirus infecting the sapindaceous weed Deinbollia borbonica in Kenya and Tanzania
Source: Arch Virol. 2017 Jan 9;162(5):1393–6. doi: 10.1007/s00705-016-3217-9 (PMC5387033; doi:10.1007/s00705-016-3217-9)
Supplement: Supplementary file 3 — Supplementary Table S1 (PDF 145 kb) [file 705_2016_3217_MOESM3_ESM.pdf]

Primers used in this study for plant species identification and virus diagnosis

Primer	Sequence (5′ → 3′)	Target	T_a (°C)
rbcLa-F[1]	ATGTCACCACAAACAGAGACTAAAGC	Chloroplast	56
rbcLa-R[1]	GTAAAA TCA AGT CCA CCR CG	Chloroplast	56
CRA/F[2a]	TGTTGACAGGTGTTTGTTTTGC	Rep	60
CRA/R[2a]	ATCACGAATTAGATCATGGCCC	MP	60
DNA-A1/F[2a]	ACGCCATTCTGTGCCTGAGGA	TrAP/REn	60
DNA-A1/R[2a]	GGACGGCGACACAACCAGCT	Rep/AC4	60
DNA-A2/F[2a]	ACTCATCGTGTCGGGAAGAGGT	CP	60
DNA-A2/R[2a]	CAAGTGAGACGCAGAGGTCCCA	Rep/TrAP	60
DNA-A3/F[2a]	TCCTGACTCGGTGCATGGGT	MP	60
DNA-A3/R[2a]	ACCTGTCCAAAATCTTGTGGGC	CP	60
DNA-A4/F[2a]	ATAGCACCGTCGGCAGAACC	Rep	60
DNA-A4/R[2a]	AGTTGCCCTCCGTTCCACCG	CR	60
CRB/F[2a]	CCTTCCATTGCTGTTGTTTGCT	CR/MP	60
CRB/R[2a]	TGGCCCAATAAAAACAAGCCCA	CR	60
DNA-B1/F[2a]	CTGACTGGGCCTGGGATGCG	MP	60
DNA-B1/R[2a]	ACTAACACACGATGCGGCTCCT	MP	60
DNA-B2/F[2a]	CTTGTGCTGCTTCCTCTTGGCT	MP	60
DNA-B2/R[2a]	GTTGCCCTCCGTTCCACCGA	CR	60
DNA-B3/F[2a]	ATGCTCGGCGATCTATAGAGGA	NSP	60
DNA-B3/R[2a]	TCAACAGCACTCGACCCAGGC	MP	60
DNA-B4/F[2a]	TTGGCTCTCAGGTGTCCACGT	CR	60
DNA-B4/R[2a]	ACGACCTCCATTACCTTCAACA	NSP	60

[1]Barcode primers used for amplification of rubisco gene for plant species identification.
[2]Overlapping primers used for generating PCR products for Sanger sequencing to validate NGS data.
[a]Primers designed for the current study.
