# Supplementary material for: Deinbollia mosaic virus: a novel begomovirus infecting the sapindaceous weed Deinbollia borbonica in Kenya and Tanzania
Source: Arch Virol. 2017 Jan 9;162(5):1393–6. doi: 10.1007/s00705-016-3217-9 (PMC5387033; doi:10.1007/s00705-016-3217-9)
Supplement: Supplementary file 4 — Supplementary Table S2 (PDF 53 kb) [file 705_2016_3217_MOESM4_ESM.pdf]

Predicted open reading frames in DNA-A and DNA-B of DMV

Isolate	Origin	Genomic component	ORF	Length (bp)	Start/Stop Codon (nt)
DB_K1	Kenya	DNA-A	V1 (CP)	777	314/1,090
		2,758 nt	V2 (MP)	351	154/504
			C1 (Rep)	1,095	1,533/2,627
			C2 (TrAP)	438	1,232/1,669
			C3 (REn)	405	1,087/1,491
			C4	240	2,213/2,452
		DNA-B	V1 (NSP)	765	457/1,221
		2,706 nt	C1 (MP)	924	1,258/2,181
DB_T1	Tanzania	DNA-A	V1 (CP)	777	315/1,091
		2,759 nt	V2 (MP)	351	155/505
			C1 (Rep)	1,095	1,534/2,628
			C2 (TrAP)	438	1,233/1,670
			C3 (REn)	405	1,088/1,492
			C4	240	2,214/2,453
		DNA-B	V1 NSP)	765	452/1,216
		2,702 nt	C1 (MP)	852	1,253/2,104
DB_T2	Tanzania	DNA-A	V1 (CP)	777	314/1,090
		2,758 nt	V2 (MP)	351	154/504
			C1 (Rep)	1,095	1,533/2,627
			C2 (TrAP)	447	1,232/1,678
			C3 (REn)	405	1, 087/1,491
			C4	240	2,213/2,452
		DNA-B	V1 NSP)	765	453/1,217
		2,702 nt	C1 (MP)	924	1,254/2,177
DB_T3	Tanzania	DNA-A	V1 (CP)	777	314/1,090
		2,758 nt	V2 (MP)	351	154/504
			C1 (Rep)	1,095	1,533/2,627
			C2 (TrAP)	438	1,232/1,669
			C3 (REn)	405	1,087/1,491
			C4	240	2,213/2,452
		DNA-B	V1 NSP)	765	453/1,217
		2,702 nt	C1 (MP)	948	1,254/2,201
